# Should the dose of tenofovir be reduced to 200–250 mg/day, when combined with protease inhibitors?

**DOI:** 10.7448/IAS.17.4.19583

**Published:** 2014-11-02

**Authors:** Andrew Hill, Saye Khoo, David Back, Anton Pozniak, Marta Boffito

**Affiliations:** 1 Pharmacology and Therapeutics, University of Liverpool, Liverpool, UK; 2 St Stephens Centre, Chelsea and Westminster Hospital, London, UK

## Abstract

**Introduction:**

The approved dose of tenofovir disproxil fumarate, 300 mg once daily, was established in clinical trials in combination with efavirenz, which does not significantly affect tenofovir concentrations. Combining tenofovir with lopinavir/r, darunavir/r or atazanavir/r increases tenofovir concentrations, which could raise the risk of renal adverse events. Newly approved tenofovir tablets are available at lower strength (200 or 250 mg) for use in paediatrics.

**Methods:**

A literature search was used to assess the effects of lopinavir/r, darunavir/r and atazanavir/r on tenofovir plasma Cmax, AUC and Cmin (Geometric Mean Ratio and 90% confidence intervals). Assuming linear dose-proportional pharmacokinetics (as observed in dose-ranging studies), the 250 mg tablet was predicted to achieve plasma concentrations 17% lower than the 300 mg dose, and the 200 mg tablet to achieve plasma levels 33% lower. Effects on tenofovir plasma Cmax, AUC and Cmin concentrations were assessed for combined dosing of each protease inhibitor with 250 or 200 mg daily doses of tenofovir, versus standard dose tenofovir (300 mg daily) without protease inhibitors.

**Results:**

In drug-drug interaction studies, lopinavir/ritonavir significantly increased tenofovir Cmax, AUC and Cmin. Effects of each PI on tenofovir Cmin were greater than effects on Cmax or AUC. Using a 250 mg paediatric dose of tenofovir with lopinavir/ritonavir, tenofovir Cmin was predicted to remain higher than tenofovir 300 mg used with efavirenz (GMR=1.26, 95% CI 1.14–1.38). Similar results were observed for use of tenofovir 250 mg with atazanavir/ritonavir (GMR=1.07, 95% CI 1.01–1.13) and with darunavir/ritonavir (GMR=1.14, 95% CI 0.99–1.31). Predicted tenofovir AUC levels for the 250 mg dose with protease inhibitors were all within the bioequivalence range, relative to use with efavirenz. Using a 200 mg paediatric dose of tenofovir with lopinavir/ritonavir, the tenofovir Cmin was predicted to be bioequivalent to tenofovir 300 mg used with efavirenz (GMR=1.02, 95% CI 0.92–1.11). Similar results were observed for use of tenofovir 200 mg with atazanavir/ritonavir (GMR=0.86, 95% CI 0.82–0.91) and with darunavir/ritonavir (GMR=0.92, 95% CI 0.80–1.05). All three results were within the bioequivalence limits of 0.8–1.25.

**Conclusions:**

Use of approved paediatric doses of tenofovir (200–250 mg once daily) in combination with lopinavir/r,darunavir/r or atazanavir/r could compensate for known drug interactions. This dose modification could potentially improve renal safety.

